# Anti-obesity Effects of *Panax ginseng*-derived exosomes via AMPK-mediated inhibition of adipocyte differentiation and lipogenesis

**DOI:** 10.1007/s13659-025-00561-4

**Published:** 2026-01-09

**Authors:** Min Ho Han, Sun Hye Lee, Youn Seon Hwang, Jong Hyun Oh, Jin Woo Kim

**Affiliations:** 1 Department of Food Technology and Nutrition, Sunmoon University, 70 Sunmoon-Ro 221 Beon-Gil, Tangjeong-Myeon, Asan-si, Chungcheongnam-Do Korea; 2 Center for Next-Generation Semiconductor Technology, Sunmoon University, 70 Sunmoon-Ro 221, Tangjeong-Myeon, Asan-si, 336-708 Chungnam Korea

**Keywords:** Anti-obesity, Lipid metabolism, AMPK signaling, *Panax ginseng*, Exosome

## Abstract

This study investigated the anti-obesity potential of *Panax ginseng*-derived exosomes (PGE) by evaluating their influence on energy metabolism, adipogenesis, and lipid accumulation. PGEs were isolated using a tangential flow filtration system, yielding particles with an average diameter of 159.5 nm and a concentration of 3.9 × 10^12^ particles/mL. In 3T3-L1 preadipocytes, PGE treatment resulted in a 72.1% reduction in lipid accumulation, as demonstrated by Oil Red O staining, indicating significant inhibition of adipogenic differentiation. Elevated expression of surface markers TET-8 (147.2%) verified the exosomal nature of the isolated vesicles. To determine their role in adipocyte differentiation, we analyzed gene and protein expression of key adipogenic markers–peroxisome proliferator-activated receptor gamma (PPAR-γ), CCAAT/enhancer-binding protein alpha and beta, and fatty acid-binding protein 4-revealing reductions of 23.6–35.6% and 26.7–35.2%, respectively. These results indicate downregulation of transcriptional and translational pathways driving adipogenesis. Lipogenic regulators, including sterol regulatory element-binding protein 1c, acetyl-CoA carboxylase, and fatty acid synthase, were also suppressed by 24.9–41.0% (gene) and 22.8–24.5% (protein), indicating impaired fatty acid synthesis. Conversely, AMP-activated protein kinase (*AMPK)* expression increased by up to 53.8% (gene) and 47.9% (protein), implying activation of energy homeostasis signaling. Immunofluorescence analysis showed a reduction in the MitoTracker/DAPI ratio (57.7–60.0%) and an increase in the F-actin/DAPI ratio (39.5–60.8%), indicating decreased mitochondrial activity and enhanced cytoskeletal integrity. These molecular changes were accompanied by AMPK activation and PPAR-γ inhibition. Collectively, these findings underscore the potential of PGEs as bioactive agents for obesity management by concurrently inhibiting adipogenesis and lipogenesis, providing a strong basis for their application in anti-obesity functional foods and pharmaceutical products.

**Graphic Abstract:**

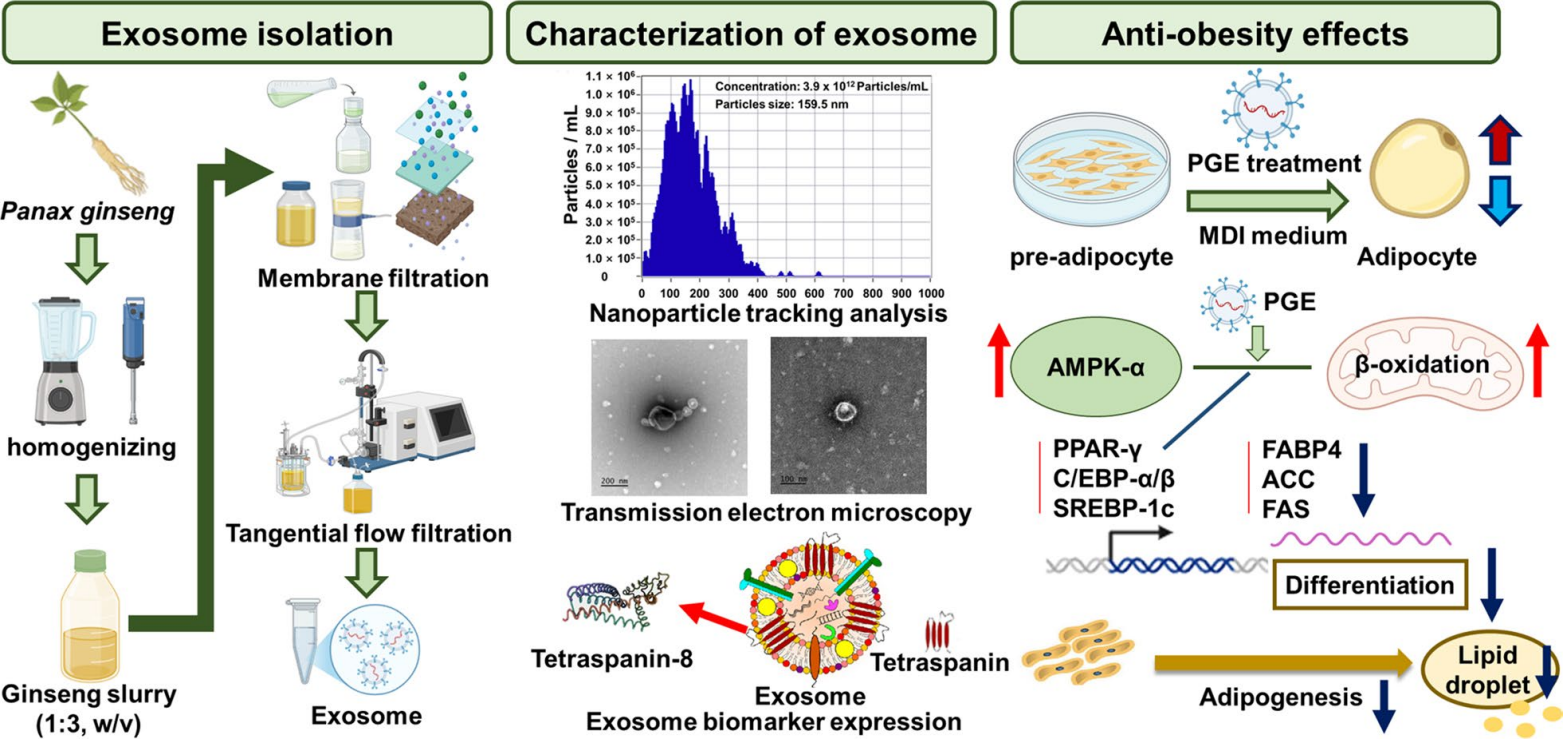

**Supplementary Information:**

The online version contains supplementary material available at 10.1007/s13659-025-00561-4.

## Introduction

Obesity is a complex metabolic disorder that develops when energy intake chronically exceeds energy expenditure, leading to excessive adipose tissue accumulation. Beyond simple weight gain, obesity involves abnormal adipocyte differentiation (adipogenesis) and lipid synthesis (lipogenesis) [1]. These processes contribute to impaired blood circulation, insulin resistance, and chronic low-grade inflammation, thereby increasing the risk of severe metabolic diseases, such as type 2 diabetes mellitus, hypertension, cardiovascular disease, nonalcoholic fatty liver disease, and certain hormone-dependent cancers [2]. According to the WHO, global adult obesity prevalence exceeded 16% in 2022, more than doubling since 1990—and continues to rise rapidly in low- and middle-income countries, underscoring obesity as a growing global public health crisis [3]. Current therapeutic strategies include dietary intervention, physical exercise, bariatric surgery, and pharmacological agents such as the lipase inhibitor orlistat, the glucagon-like peptide-1 (GLP-1) receptor agonist liraglutide, and the appetite suppressant phentermine. However, these drugs are associated with adverse effects, including steatorrhea, increased heart rate, gastrointestinal discomfort, insomnia, and reduced tolerability, which limit their long-term use [4].

The pathogenesis of obesity is driven by complex interactions among transcription factors and signaling pathways that regulate adipocyte differentiation and lipid metabolism. Disruption of systemic energy homeostasis promotes excessive triglyceride deposition in adipose tissue and increases the adipocyte number. Adipocyte differentiation is primarily controlled by the upregulation of key transcription factors, such as peroxisome proliferator-activated receptor gamma (PPAR-γ) and CCAAT/enhancer-binding protein alpha (C/EBPα), which activate the transcription of lipid storage-related genes, including fatty acid-binding protein 4 (*FABP4*) and fatty acid synthase (*FAS*) [5]. During early differentiation, C/EBP-β induces the expression of PPAR-γ and C/EBP-α, promoting adipocyte maturation. Sterol regulatory element-binding protein-1c (SREBP-1c) regulates fatty acid biosynthetic enzymes, such as FAS and acetyl-CoA carboxylase (ACC), directly contributing to lipogenesis. Conversely, AMP-activated protein kinase (AMPK) becomes activated under energy-deficient conditions in response to a low ATP/AMP ratio, promoting fatty acid oxidation and suppressing lipid synthesis in adipose tissue, liver, and muscle, thereby restoring energy balance [6]. A mechanistic understanding of adipocyte differentiation and fatty acid biosynthesis is fundamental for elucidating the pathophysiology of obesity and developing targeted therapeutic strategies using metabolic regulators, like AMPK. Given the limitations of synthetic drugs, there is growing interest in natural bioactive compounds as anti-obesity agents, owing to their high biocompatibility, favorable safety profiles, and reduced side effects during long-term use. In particular, natural products targeting the AMPK pathway—a pivotal regulator of adipogenesis and lipid metabolism—are actively being explored as sustainable and safe therapeutic options.

*Panax ginseng*, first documented in the Chinese medical text *Shennong Bencao Jing* during the Han Dynasty, has been used for millennia in East Asia as a medicinal herb. It contains a wide range of bioactive constituents, including ginsenosides, flavonoids, and polysaccharides [7]. Preclinical studies indicate that ginseng extracts downregulate PPAR-γ, C/EBP-α, and SREBP-1c in 3T3-L1 preadipocytes, inhibit fatty acid biosynthetic enzymes such as ACC, FAS, and ATP citrate lyase (ACL), and thereby reduce lipid accumulation [8]. Despite these bioactivities, the clinical potential of ginseng-derived compounds is limited by poor bioavailability and inadequate tissue-targeting, resulting from low membrane permeability, instability, and rapid metabolism. To overcome these pharmacokinetic constraints, plant-derived exosome (PDE)-based delivery systems have emerged as a promising strategy. Exosomes are extracellular vesicles with a lipid bilayer structure (30–150 nm in diameter) that encapsulate biomolecules such as mRNAs, microRNAs, and proteins, playing key roles in intercellular communication and gene regulation [9]. PDEs exhibit advantageous properties, including low immunogenicity, gastric stability, and biodegradability, making them suitable for oral delivery. Recent studies have reported that ginseng-derived nanovesicles exert diverse physiological effects, such as anti-inflammatory, pro-survival, and anti-tumor activities [10].

This study aimed to elucidate the molecular mechanisms by which *Panax ginseng*-derived exosomes (PGEs) modulate adipocyte differentiation, lipid accumulation, and fatty acid biosynthetic pathways in 3T3-L1 cells, thereby exerting anti-obesity effects. Adipocyte differentiation is primarily driven by the transcriptional activation of PPAR-γ and C/EBP-α, which subsequently induce SREBP-1c and upregulate key fatty acid biosynthetic enzymes, such as ACC, FAS, and ACL, ultimately leading to intracellular lipid accumulation [11]. In this study, we investigated whether PGEs suppress the expression of these master transcription factors and metabolic enzymes, activate the AMPK signaling pathway, and preserve mitochondrial function, thereby creating a metabolic environment that counteracts adipogenesis and lipogenesis. By delineating these molecular events, this study seeks to establish PGEs as a functional exosomal system capable of directly targeting key metabolic pathways in adipocytes and to provide a scientific basis for its potential application in developing natural product-based anti-obesity therapeutics for nutraceutical and pharmaceutical use.

## Methods and materials

### Exosomes isolation

Fresh six-year-old *P. ginseng* (cv. Sunil) cultivated in Eumseong, Chungcheongbuk-do, Korea, was used in this study, and for exosome isolation, ginseng was mixed with phosphate-buffered saline (PBS) at a ratio of 1:3 (w/v) and homogenized to obtain a uniform slurry, after which the homogenate was centrifuged at 2000 × g for 30 min to remove coarse particles and fibrous materials, followed by centrifugation at 10,000 × g for 60 min to eliminate cellular debris and insoluble solids, and the clarified supernatant was then sequentially filtered through membrane filters with pore sizes of 1.22 μm, 0.45 μm, and 0.22 μm (Hyundai Micro Co., Ltd., Seoul, Korea) to remove fine particles and residual impurities, and finally, for purification and concentration of PGE, tangential flow filtration (TFF) was conducted using a 100 kDa molecular weight cut-off (MWCO) Minimate™ TFF Capsule equipped with an Omega™ membrane (Cytiva, Marlborough, MA, USA) and operated with a Masterflex^®^ L/S^®^ Digital Drive and an Easy-Load^®^ II pump head (Masterflex, Avantor, Vernon Hills, IL, USA) at a feed flow rate of 40.0 mL/min. All procedures were conducted under aseptic conditions using sterilized reagents and equipment, and the final exosome suspensions were filtered through a 0.22 μm sterile membrane to ensure sterility.

### Characterization of exosomes

The particle size and concentration of PGE were measured using nanoparticle tracking analysis (NTA) with a ZetaView instrument (PMX 130, Particle Metrix GmbH, Meerbusch, Germany). NTA operates by tracking the Brownian motion of individual nanoparticles dispersed in a liquid under illumination with a 488 nm laser, enabling determination of particle size distribution and concentration from scattered light intensity. To minimize measurement errors caused by particle overlap and multiple scattering, PGE was diluted 1:10 (v/v) in ultrapure water pre-filtered through a 0.1 μm membrane filter (Hyundai Micro Co., Ltd., Seoul, Korea). Particle size distribution was assessed at 11 distinct positions within the sample based on particle diameter, and the particle count in each fraction was quantified. The particle size was calculated from the diffusion coefficient according to the Stokes–Einstein equation, and the particle concentration was corrected by applying the dilution factor. To confirm that the isolated particles were exosomes, the expression of the plant-specific tetraspanin exosome-associated marker TET8 was evaluated.

### Culture and differentiation of 3T3-L1 preadipocytes

3T3-L1 preadipocytes, which are widely utilized as a well-characterized in vitro model for adipogenesis and lipid metabolism research, were obtained from the Korean Cell Line Bank (Seoul, Korea) and maintained in Dulbecco’s Modified Eagle Medium (DMEM) supplemented with 10.0% (v/v) newborn bovine calf serum (NBCS) and 1.0% (v/v) penicillin at 37.0°C in a humidified atmosphere containing 5.0% CO₂ using a cell incubator (SA-MCO-18AIC, Sanyo Co., Osaka, Japan). Differentiation of preadipocytes into mature adipocytes was induced by replacing the culture medium with DMEM containing MDI (0.5 mM isobutylmethylxanthine, 1.0 mM dexamethasone, and 1.0% insulin) and 5.0% (v/v) fetal bovine serum (FBS) every 48 h for a total of four cycles. All reagents used for cell culture and analysis were of analytical grade or higher quality and were purchased from Thermo Fisher (Waltham, MA, USA).

### Cell activity assay

Cell activity was assessed based on the reduction of 3-(4,5-dimethylthiazol-2-yl)-2,5-diphenyltetrazolium bromide (MTT). To evaluate changes in cell activity, 3T3-L1 cells cultured under identical conditions were treated with PGE (0.0–3.9 × 10^12^ particles/mL) and incubated for an additional 24 h. Subsequently, 0.6 μM MTT solution was added and incubated for 4 h, after which the resulting formazan crystals were dissolved in dimethyl sulfoxide. The absorbance was measured at 450 nm using a microplate reader (AMR-100, Allsheng, Hangzhou, Zhejiang, China), and cell activity was expressed as a percentage according to Eq. 1.

1 $$\text{Cell activity }\left(\text{\%}\right)\text{ = }\left( {1} - \frac{\text{Absorbance of PGE}-\text{treated group}}{\text{Absorbance of untreated control group}} \right) \times {100}$$

### Lipid accumulation assay

Lipid accumulation is characterized by an increase in both the size and number of lipid droplets, which are spherical non-membranous organelles formed by the intracellular deposition of neutral lipids, and serves as a representative indicator of adipogenic differentiation in preadipocytes such as 3T3-L1 cells [12]. The extent of lipid accumulation was quantified by Oil Red O staining, which selectively stains intracellular neutral triglycerides accumulated during differentiation. After treatment of 3T3-L1 cells with PGE (0.0–9.8 × 10^10^ particles/mL) and an additional 24 h of incubation, cells were fixed with 10.0% (v/v) formaldehyde for 30 min and stained with Oil Red O solution for 60 min. The morphology of lipid droplets and the degree of neutral lipid accumulation were visualized under an optical microscope (CX43, Olympus, Tokyo, Japan), and the bound dye was subsequently eluted with 99.5% (v/v) isopropanol. The absorbance was measured at 497 nm, and lipid accumulation was expressed as a percentage according to Eq. 2.

2 $${\text{Lipid accumulation }}\left( {\text{\% }} \right) = \left( {1 - \frac{{{\text{Absorbance of PGE}} - {\text{treated group}}}}{{\text{Absorbance of untreated control group}}}} \right)\;{\kern 1pt} \times \;100$$

### Gene expression analysis

To examine the effects of PGE on adipogenic differentiation and fatty acid synthesis, total RNA was extracted from PGE-treated 3T3-L1 cells and changes in gene expression were evaluated. The experimental groups included a non-treated undifferentiated group (N.T.), a fully differentiated control group (DC), and PGE-treated groups at different concentrations. Total RNA from each group was isolated using the AccuPrep^®^ RNA Extraction Kit (Bioneer, Daejeon, Korea). Subsequently, reverse transcription was performed with 1.0 μg of RNA using the cDNA Synthesis Platinum Master Mix (GenDEPOT, Barker, USA), and quantitative reverse transcription polymerase chain reaction (qRT-PCR) was conducted to quantify the expression of key target genes (Table 1). Gene expression levels were quantified after electrophoresis on 1.5% (w/v) agarose gels containing GelRed^®^ nucleic acid stain (Komabiotech, Seoul, Korea) and analyzed using the Davinch-Gel™ Imaging System (Young In Labplus, Seoul, Korea) together with CLIQS software (TotalLab, UK).

**Table 1:** Primer sequences of major genes used in qRT-PCR analysis for evaluation of gene expression of anti-obesity in 3T3-L1

Gene	Forward (5′-3′)	Reverse (5′-3′)
^*1)*^*PPAR-γ*	GACACCAGTTTTGCCTCCAGTA	TCCAGAGGCGGAAGTTCTGT
^2)^*C/EBP-α*	CCCACCAACTTCGGAATCAG	CCATTGGGTCAGCTCTTGTGA
^3)^*C/EBP-β*	TGCGAGCACGAGACGTCTAT	GCCAGGAACTCGTCGTTGAA
^*4)*^*FABP4*	AAGGTGAAGAGCATCATAACCCT	TCCACAAGTCACGCCTTTCATA
^*5)*^*SREBP-1c*	GCCCTACGGGTCAAAACCA	ACGCGCACTGCTCAAAAAC
^6)^*FAS*	GAAGCTTGGGTCATGAATGAACT	CGATTGACTTCCCTGCTTGAA
^7)^*ACC*	GAGGCTCCTGGGTGGTCATA	GTCCTCGCGAGCCTTTAGC
^8)^*AMPK-α*	TCCTGCTTGATGCACACATGA	CCTCGTGGCCTGTGGCCATT
^9)^*GAPDH*	GATGGGCATGAAGCATGAGA	TGGCATGGACTGTGGTCATT

^*1*^^)^*PPAR*-*γ*: peroxisome proliferator-activated receptor gamma, ^*2)*^*C/EBP-α*: CCAAT/enhancer-binding protein alpha, ^3)^*C/EBP-β*: CCAAT/enhancer-binding protein beta, ^4)^*FABP4*: fatty acid-binding protein 4, ^5)^*SREBP-1c*: sterol regulatory element binding protein-1c, ^6)^*FAS*: fatty acid synthase, ^7)^*ACC*: Acetyl-CoA carboxylase, ^8)^*AMPK-α*: AMP-activated protein kinase, ^9)^*GAPDH*: glyceraldehyde 3-phosphate dehydrogenase

### Protein expression analysis

To evaluate the expression of proteins associated with adipogenic differentiation and fatty acid synthesis, total protein was extracted from PGE-treated 3T3-L1 cells (0.0–9.8 × 10^10^ particles/mL) using RIPA buffer supplemented with a protease inhibitor cocktail. Protein concentrations were determined using the Bradford assay, after which 30.0 μg of protein was separated by electrophoresis on a 12.0% SDS–polyacrylamide gel and subsequently transferred onto a polyvinylidene difluoride (PVDF) membrane. To block non-specific binding, the membrane was incubated with 5.0% (w/v) non-fat dry milk. Primary and secondary antibodies, including those against exosome-specific biomarkers, were diluted 1:100–1:2000 in 5.0% (w/v) non-fat dry milk and incubated with the membrane. Protein expression was detected using enhanced chemiluminescence (ECL) and quantified using the Kwik Quant Pro Multi-Image System (D1010, Kindle Bio, Greenwich, CT, USA).

### Immunofluorescence analysis

To evaluate the inhibitory effects of PGE on adipogenic differentiation in 3T3-L1 cells, nuclear morphology, mitochondrial activity, and cytoskeletal organization were assessed by immunofluorescence analysis. 3T3-L1 preadipocytes maintained under identical culture conditions were treated with PGE and subsequently incubated for an additional 24 h, followed by fixation with 4.0% paraformaldehyde for 30 min. The cells were then permeabilized with 0.1% Triton X-100 for 10 min and blocked with 0.2% BSA to prevent nonspecific binding. For cytoskeletal and mitochondrial staining, cells were incubated with Phalloidin-iFluor™ 488 (F-actin) and MitoTracker™ Green FM in the dark at 4.0°C, followed by incubation with Alexa Fluor® 594–conjugated secondary antibody at room temperature (RT) in the dark. Finally, nuclear DNA was counterstained with 4′,6-diamidino-2-phenylindole (DAPI), and fluorescence signals were acquired and quantitatively analyzed for distribution and relative intensity using a CKX53 fluorescence microscope (Olympus, Shinjuku, Tokyo, Japan) equipped with FITC/TRITC filters.

### Flow cytometric analysis of AMPK-α expression

AMPK-α expression was quantified at the single-cell level by flow cytometry to evaluate the metabolic regulatory effects of PGE. 3T3-L1 preadipocytes were seeded at 1.0 × 10^5^ cells/well in 6-well plates and cultured for 24 h. Control groups included N.T. and DC, while treatment groups were exposed to 0.0–3.8 × 10^10^ particles/mL PGE for an additional 24 h. Cells were harvested with trypsin–EDTA, washed twice with cold PBS, and centrifuged at 300 × g for 5 min. Fixation was performed with 4.0% paraformaldehyde (15 min, RT), followed by permeabilization with 0.1% saponin in PBS containing 1.0% BSA (10 min). Non-specific binding was blocked with 1.0% BSA/PBS for 30 min, after which cells were incubated with AMPK alpha-1 Monoclonal Antibody (Thermo Fisher, 1:300) for 30 min at RT in the dark. After washing, cells were incubated with goat anti-mouse IgG Alexa Fluor 488 secondary antibody (Thermo Fisher, 1:1,000) for 30 min at RT. Fluorescence signals were acquired on a CytoFLEX V0-B4-R0 (Beckman Coulter, Brea, CA, USA) using the 488 nm (FITC/AF488) channel and analyzed with CytExpert v2.5.0.77. Data were quantified as mean fluorescence intensity (MFI) and percentage of AMPK-α-positive cells. Gating was based on FSC/SSC parameters with doublets excluded by FSC-A/H, and a minimum of 10,000 events was collected per sample.

### Quantitative and qualitative analysis of ginsenosides

For qualitative and quantitative analysis of ginsenosides in PGE, the samples were filtered through a 0.22 μm membrane filter (Hyundai Micro Co., Ltd., Seoul, Korea) and analyzed using a Vanquish Flex ultra-high-performance liquid chromatography (UHPLC) system (Thermo Fisher Scientific) coupled with a TSQ Altis Plus mass spectrometer (Thermo Fisher) equipped with a ROC C18 column (3.0 × 150.0 mm, particle size 3.0 μm, Restek Ltd., PA, USA). The mobile phases consisted of HPLC-grade acetonitrile (JT Baker, Phillipsburg, NJ, USA) and Milli-Q® ultrapure water (Milli-Q^®^ IQ7000, Sigma Aldrich), each containing 0.1% (v/v) formic acid (Alfa Aesar, Haverhill, USA). Chromatographic separation was performed under the following linear gradient program: 0–5 min, 20.0% acetonitrile; 5–35 min, 20.0–80.0% acetonitrile; 35–40 min, 80.0% acetonitrile, followed by re-equilibration to the initial condition. The flow rate was maintained at 0.3 mL/min, the injection volume was set at 5.0 μL, and the column temperature was maintained at 40°C. Mass spectrometric detection was carried out in electrospray ionization (ESI) negative mode using multiple reaction monitoring (MRM), with major MRM transitions and collision energies optimized by analysis of authentic standards. The MS source parameters were as follows: spray voltage, 3.5 kV; capillary temperature, 320℃; sheath gas, 35.0 arbitrary units (arb); and auxiliary gas, 10.0 arb. Qualitative identification of ginsenosides was based on retention time (R.T.), mass-to-charge ratio (m/z), characteristic fragment ions, ion ratios (qualifier/quantifier transitions), and peak areas, whereas quantitative analysis was performed using calibration curves (10.0–500.0 ng/mL) constructed from authentic ginsenoside standards (≥ 98.0% purity, Sigma Aldrich) with normalization to an internal standard based on peak-area ratios.

### Statistical analysis

All experiments were performed in triplicate, and the results are expressed as the mean ± standard deviation. Statistical analyses were conducted using GraphPad Prism software version 10.4.1 (GraphPad Software, San Diego, CA, USA). Statistical significance was determined at a threshold of *p* < 0.05 using one-way analysis of variance (ANOVA) and Student’s *t*-test.

## Results and discussions

### Characterization of exosomes

**Fig. 1:**
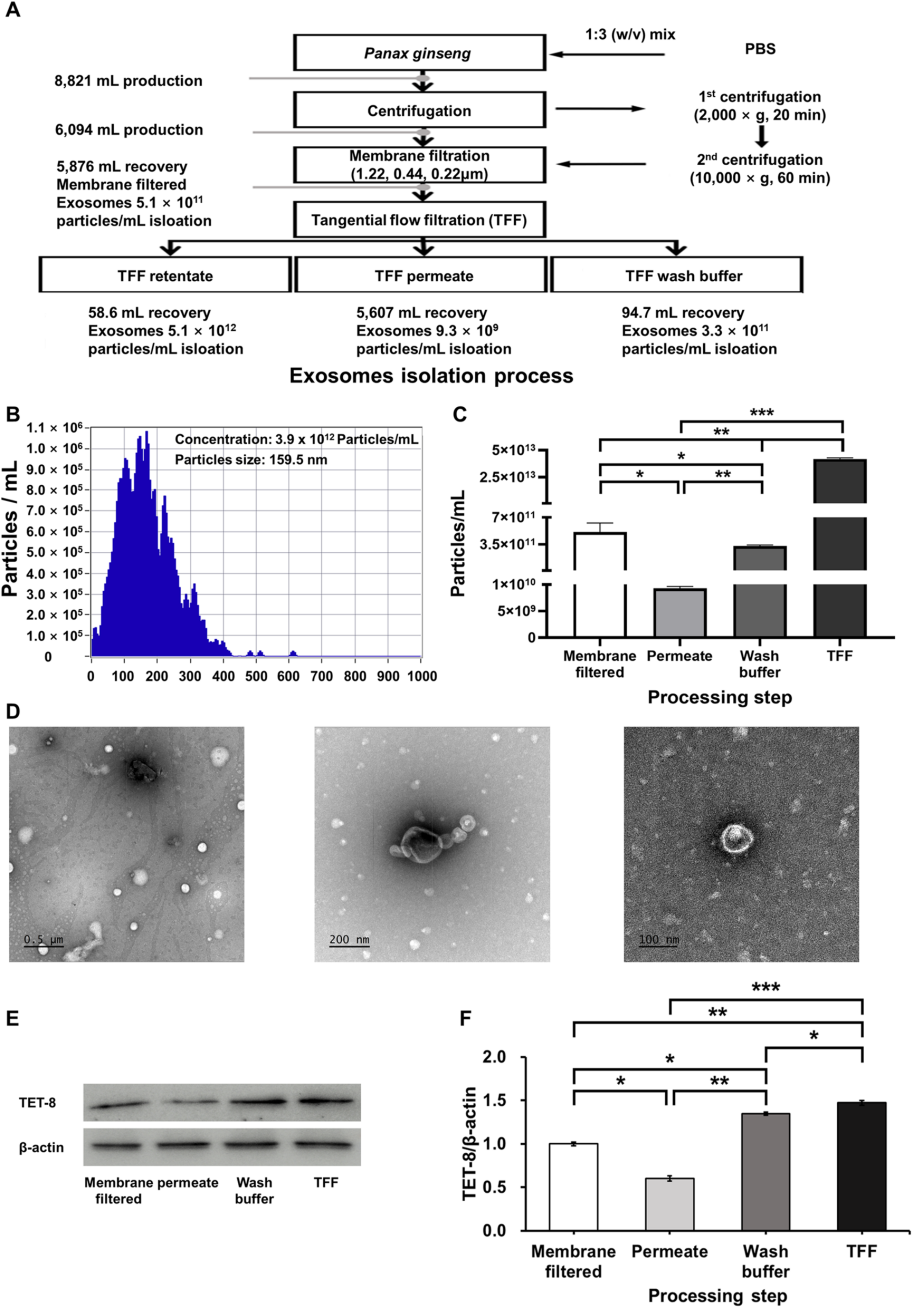
Sequential membrane filtration and tangential flow filtration (TFF)-based purification of *Panax ginseng*-derived exosomes. (**A**) Particle size distribution and concentration measured using nanoparticle tracking analysis. (**B**) Quantification of exosome yield at each purification step. (**C**. Transmission electron microscopy images of *P. ginseng*-derived exosomes. (**D**) **E** Evaluation of biomarker expression by Western blot. Quantification of **F** TET-8 expression levels in *P. ginseng*-derived exosomes

Exosomes are nanosized extracellular vesicles that play a central role in intercellular communication and the regulation of physiological processes. They have recently attracted attention as potential diagnostic biomarkers and drug delivery vehicles for various diseases [13]. However, for effective in vivo application, it is critical to establish reliable methods that ensure high-purity and high-yield isolation, as these are prerequisites for maintaining bioactivity and ensuring standardized quality control. In the present study, sequential membrane filtration was integrated with a tangential flow filtration (TFF)-based continuous purification system to isolate and purify PGEs from ginseng slurry (Fig. 1A; Table 2). Subsequently, qualitative, quantitative, and physicochemical analyses of the purified PGEs were performed using nanoparticle tracking analysis and biomarker expression profiling.

The results revealed that the mean diameter of PGEs isolated from ginseng slurry was 159.5 nm, which falls within the typical size range of exosomes (50.0–200.0 nm) (Fig. 1B). Furthermore, light scattering analysis using the ZetaView system confirmed a uniform population of spherical particles, verifying that the isolated vesicles were nanoscale particles exhibiting the morphological characteristics of exosomes. To confirm this, transmission electron microscopy (TEM) analysis clearly revealed the characteristic morphology of exosomes, showing spherical or cup-shaped vesicles with intact bilayered membranes (Fig. 1D). The concentration of PGE was 3.9 × 10^12^ particles/mL, which was 4.8 × 10^2^–1.1 × 10^5^-fold higher than that reported for *Solanum nigrum L.* exosomes obtained via polyethylene glycol (PEG) precipitation (8.2 × 10^9^), *Aloe vera* exosomes obtained via ultrafiltration (6.5 × 10^8^), and *Zingiber officinale* exosomes obtained via ultracentrifugation (3.6 × 10^7^) [14–16]. Also, compared with PGE isolated by conventional ultracentrifugation (4.18 × 10^11^ particles/mL) and polyethylene glycol precipitation (1.09 × 10^12^ particles/mL), the purified PGE obtained in this study exhibited 9.3-fold and 3.6-fold higher concentrations, respectively [17, 18]. Moreover, compared with the exosome concentration obtained from sequential membrane filtration alone (5.1 × 10^11^ particles/mL) and from TFF filtrate (9.3 × 10^9^ particles/mL), the purified PGE concentration was about 8-fold and 420-fold higher, respectively (Fig. 1C). These findings indicate that the applied isolation process achieved high-efficiency, exosomes-specific recovery, yielding high concentrations without inducing physical damage to the vesicles. In addition, analyses of the filtrate and wash fractions revealed that they contained only 1.7% and 1.0% of the total recovered exosomes, respectively, corresponding to an overall process loss rate of 12.3%. This figure suggests that the pore size and flow rate parameters of the selected membranes effectively minimized nonspecific loss.

To confirm the identity of the isolated nanoparticles as exosomes, we examined the expression levels of the plant-specific tetraspanin protein TET-8, which serves as a key marker for plant-derived extracellular vesicles. Consistent with previous reports, the ginseng-derived vesicles exhibited positive expression of TET8, confirming their exosomal characteristics and validating TET8 as a reliable marker for plant-derived extracellular vesicles. TFF-purified PGEs exhibited a 1.5-fold higher TET8 expression than membrane-filtered exosomes, confirming that TFF purification enhances the enrichment of exosomal components (Fig. 1E, F) [19]. These findings provide clear evidence that the isolated nanoparticles were plant-derived exosomes. Collectively, the results emphasize the advantages of the TFF-based continuous purification process over conventional PEG precipitation, ultracentrifugation, and immunoaffinity chromatography in achieving higher yield and purity. Therefore, the membrane filtration–TFF-based isolation method established in this study offers notable benefits in terms of structural stability, bioactivity preservation, and recovery efficiency, suggesting strong potential for industrial-scale production and functional material development. Furthermore, compared with animal cell-derived exosomes, plant-derived exosome offer inherent advantages, including superior safety, low production costs, and sustainability, underscoring their promise as a next-generation biotechnological platform.

### Cell activity assay

Cell activity is a critical parameter for quantitatively assessing the influence of a substance on cellular metabolic function, thereby serving as a key indicator for evaluating physiological safety and biocompatibility. In particular, assessing cell activity at the preadipocyte stage provides a fundamental basis for screening the safety and efficacy of potential functional materials during the preclinical phase [20]. In this study, we evaluated the activity of 3T3-L1 cells following treatment with varying concentrations of PGEs, aiming to determine the optimal concentration that minimizes interference with bioactivity-related mechanisms during subsequent anti-obesity evaluations.

**Fig. 2:**
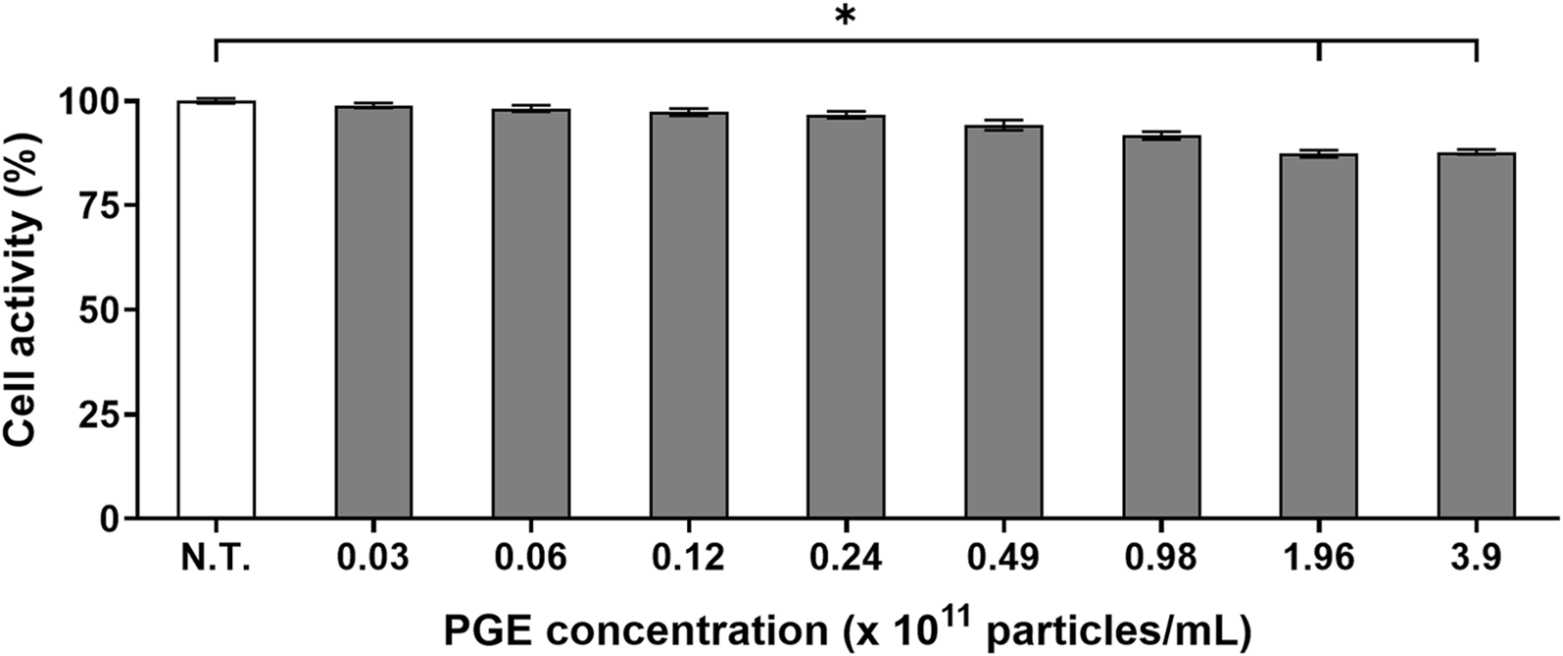
Effect of *Panax ginseng*-derived exosome on 3T3-L1 preadipocyte activity by MTT assay

The evaluation of metabolic activity in 3T3-L1 cells treated with varying concentrations of PGEs (0.0–3.9 × 10^12^ particles/mL) revealed that treatment at 0.98 × 10^11^ particles/mL maintained cell viability at 91.6% relative to the non-treated (N.T.) control, with no statistically significant reduction (*p* > 0.05, Fig. 2). In contrast, concentrations ≥ 1.96 × 10^11^ particles/mL exhibited a downward trend in cell activity (≤ 87.3%), which likely reflects metabolic pathway reprogramming induced by high-dose bioactive substances [21]. Previous studies have reported that elevated concentrations of plant-derived bioactive compounds can alter intracellular transcription, protein translation, and energy metabolism through mechanisms such as excessive activation of the AMPK pathway, autophagy induction via mTORC1 inhibition, or downregulation of SREBP-1c signaling to inhibit lipid biosynthesis [22]. These regulatory events often redirect cellular metabolism toward energy conservation or downregulate specific metabolic functions while preserving survival capacity, which may manifest as transient reductions in MTT assay-based activity. Accordingly, it is plausible that bioactive constituents within PGE may modulate cellular responsiveness under high-concentration conditions. Therefore, in this study, we selected a concentration ≤ 9.8 × 10^10^ particles/mL for subsequent experiments, as this level maintained stable cell activity while ensuring interpretive accuracy of bioactivity and mechanistic analyses. This approach enabled us to reliably elucidate the mechanism of action of PGEs without the confounding effects of nonspecific activity reduction.

This finding indicates that PGEs possess high biocompatibility, with minimal disruption to cellular function and metabolic activity. Collectively, these results highlight the potential of PGEs as a natural product-derived extracellular vesicle that can maintain basal cellular homeostasis while exerting bioactivity, offering significant advantages in biological activity retention and safety over conventional synthetic anti-obesity agents.

### Assessment of lipid accumulation

Adipocyte differentiation is primarily governed by the activation of key transcription factors such as PPAR-γ and C/EBP-α, whose elevated expression facilitates adipocyte maturation, lipid droplet formation, and triglyceride accumulation [23]. Lipid droplets serve as major intracellular organelles for lipid storage and mobilization, and their size and density are strongly linked to lipid accumulation and metabolic homeostasis. These features are widely employed as pathological indicators of obesity and related metabolic disorders [24]. In this study, to elucidate the effects of PGEs on lipid droplet formation during differentiation, 3T3-L1 preadipocytes were induced to differentiate, and neutral lipid deposition was quantitatively assessed using Oil Red O staining.

**Fig. 3:**
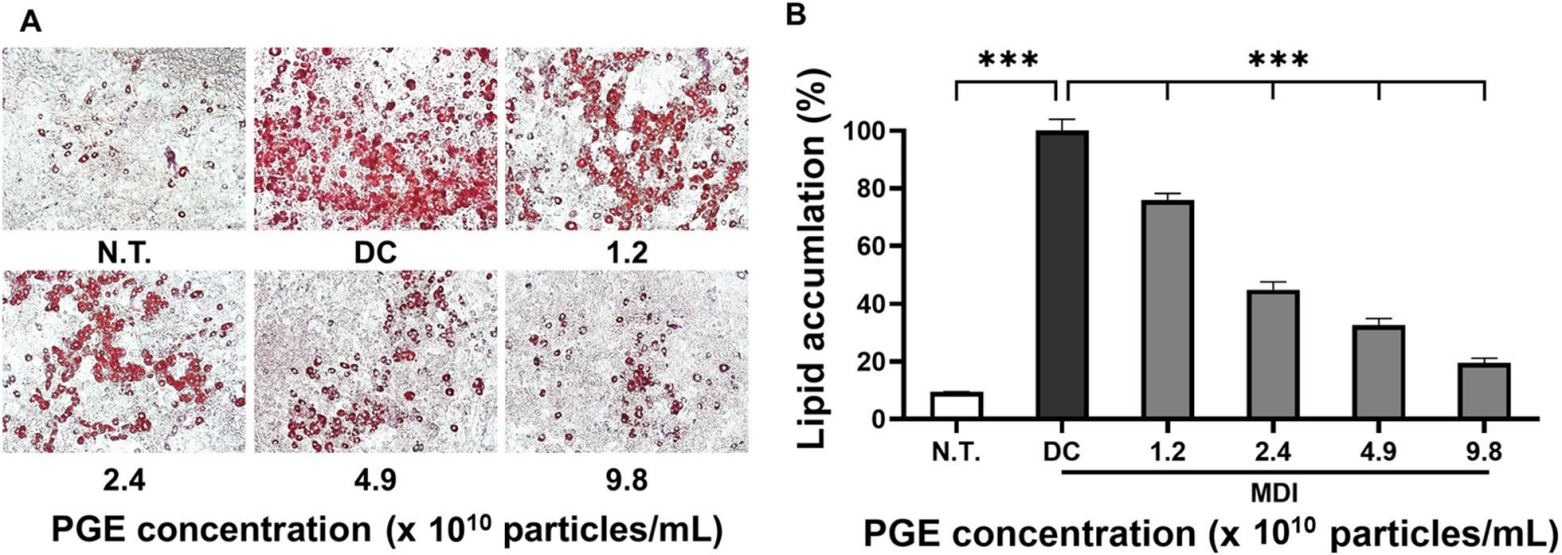
Effect of *Panax ginseng*-derived exosomes on preadipocyte differentiation and lipid accumulation of 3T3-L1 preadipocyte. (**A**) Oil Red O staining of intracellular lipid droplets following treatment with *P. ginseng*-derived exosomes. (**B**) Quantification of lipid accumulation relative to control

As shown in Fig. 3, the differentiated control (DC) group exhibited substantial lipid droplet formation, with overall lipid accumulation increasing by 278.3% compared to the N.T. control. This was accompanied by marked enlargement in droplet size and density. In contrast, PGE-treated groups demonstrated a progressive reduction in lipid accumulation, with the highest concentration group showing only fine droplets or droplets of relatively low-density. Quantitative analysis (Fig. 3B) revealed that lipid accumulation in the PGE-treated groups decreased by 23.4%, 36.7%, and 63.5% at 1.2 × 10^10^particles/mL, 2.4 × 10^10^particles/mL, and 4.9 × 10^10^ particles/mL, respectively, when compared with the DC group, confirming a significant dose-dependent inhibitory effect (*p* < 0.01). Notably, treatment with PGEs at 9.8 × 10^10^ particles/mL produced a 64.7% reduction in lipid accumulation, representing the strongest inhibitory effect observed, which was attributed to the suppression of fatty acid synthesis and adipocyte differentiation, thereby inhibiting lipid droplet formation.

The suppression of lipid accumulation and droplet formation may help normalize energy storage capacity and maintain metabolic balance in adipose tissue, which in turn could contribute to long-term reduction of body fat and prevention of obesity [25]. The observed decrease in lipid accumulation indicates that PGEs markedly inhibited lipid droplet formation during 3T3-L1 differentiation, likely through the downregulation of key transcription factors involved in adipogenesis, including PPAR-γ, C/EBP-α, and C/EBP-β. This downregulation may subsequently suppress fatty acid synthesis and triglyceride deposition [26]. Furthermore, these findings highlight the potential of PGEs as a functional material with anti-obesity activity by regulating lipid metabolism and underscore the need for follow-up studies involving gene and protein expression analyses to further clarify their mechanism of action.

### Expression analysis of adipogenic and lipogenic factors

Adipocyte differentiation is mediated by the coordinated regulation of multiple transcription factors and signaling pathways that collectively induce fatty acid biosynthesis and triglyceride accumulation—key molecular mechanisms contributing to obesity [27]. Among these, PPAR-γ and the C/EBP family of transcription factors play essential roles in initiating and promoting the early differentiation and maturation of preadipocytes. FABP4 functions as a key mediator of intracellular fatty acid transport and storage, thereby contributing significantly to lipid metabolism. Similarly, SREBP-1c, FAS, and ACC are well-recognized regulators of fatty acid synthesis and lipid accumulation pathways [28]. In this study, we performed a comprehensive analysis of the effects of PGEs on both transcriptional and translational levels of major regulators associated with adipocyte differentiation and lipid metabolism in 3T3-L1 cells, including AMPK-α, to elucidate the molecular mechanisms underlying their anti-obesity effects.

**Fig. 4:**
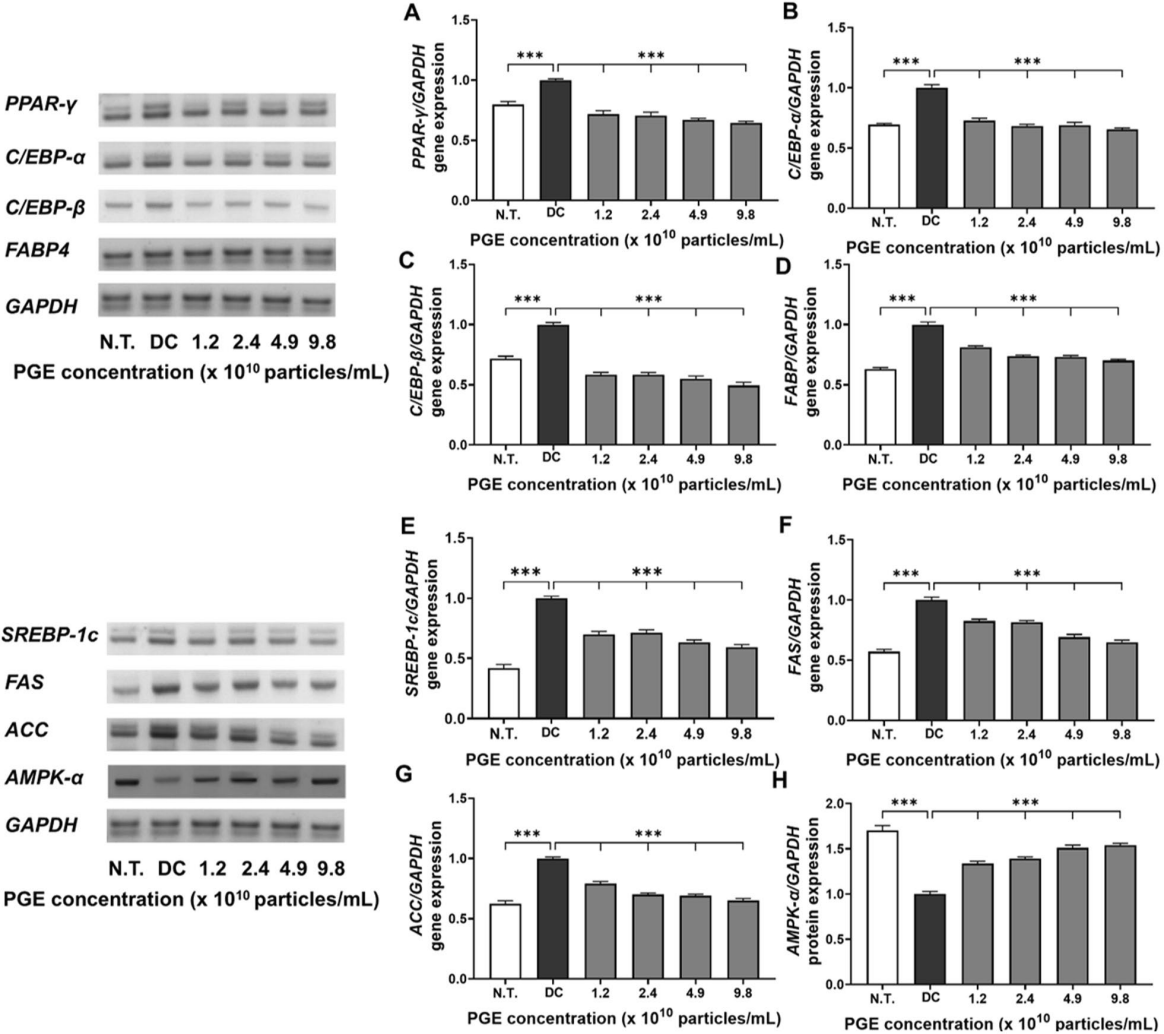
Evaluation of anti-obesity related gene expression in 3T3-L1 following treatment with *Panax ginseng*-derived exosome. Expression levels of *PPAR-γ* (**A**), *C/EBP-α* (**B**), *C/EBP-β* (**C**), *FABP4* (**D**), *SREBP-1c* (**E**), *FAS* (**F**), *ACC* (**G**), and *AMPK-α* (**H**) gene levels, quantified relative to *GAPDH*

**Fig. 5:**
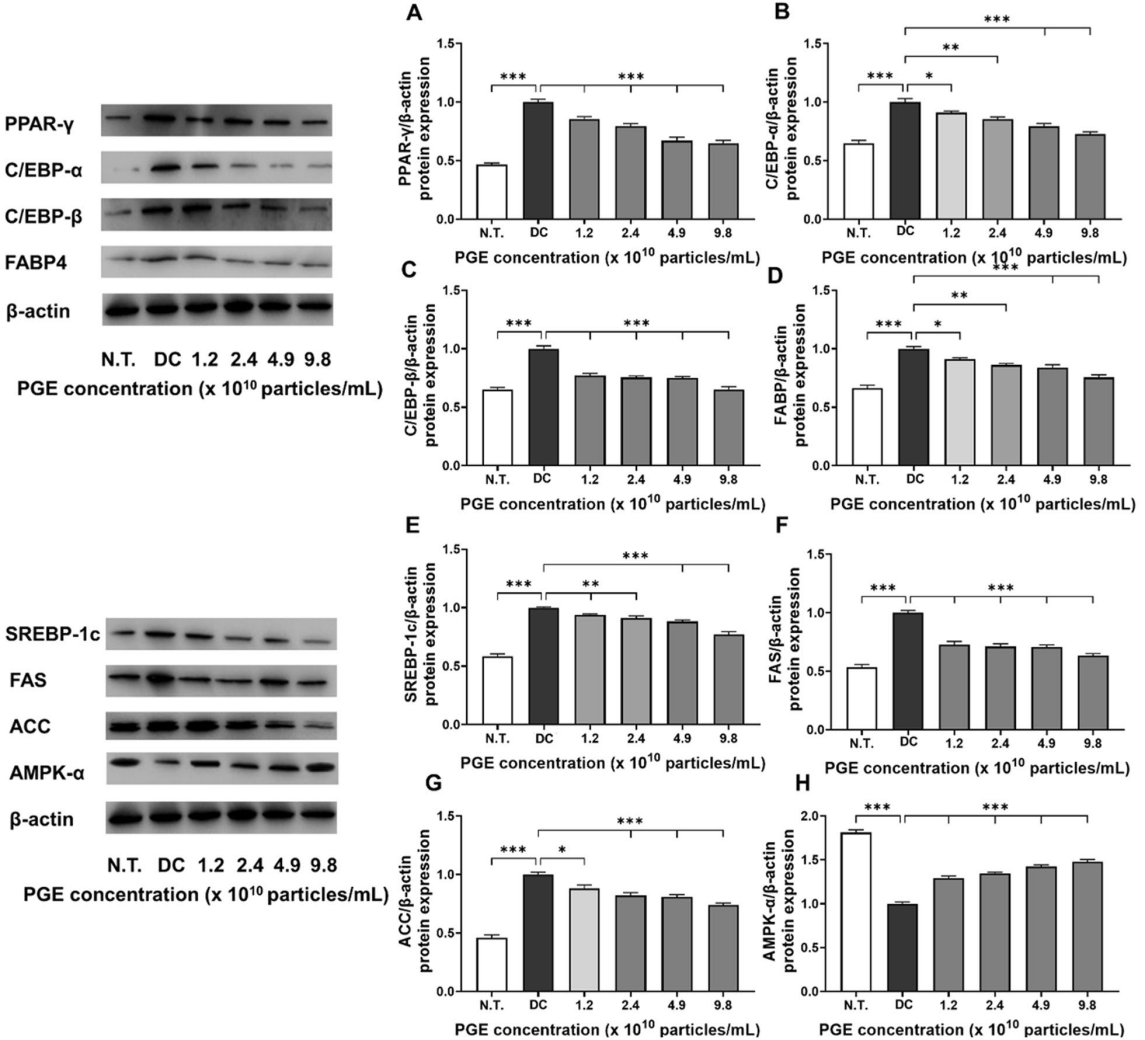
Evaluation of anti-obesity related protein expression in 3T3-L1 following treatment with *Panax ginseng*-derived exosome. Expression levels of PPAR-γ (**A**), C/EBP-α (**B**), C/EBP-β (**C**), FABP4 (**D**), SREBP-1c (**E**), FAS (**F**), ACC (**G**), and AMPK-α (**H**) protein levels, quantified relative to β-actin

As illustrated in Fig. 4A–D and Fig. 5A–D, the expression of adipogenesis-promoting factors was markedly higher in the DC group compared to the N.T. group, whereas PGE treatment significantly attenuated these levels. Specifically, the mRNA and protein levels of PPAR-γ were reduced by 28.3–35.6% and 14.5–35.2%, respectively, in the PGE-treated groups relative to the DC group. Similarly, gene expression of C/EBP-α and C/EBP-β decreased by 23.6–27.1% and 21.7–29.9%, while their protein levels dropped by 23.8–34.7% and 9.0–27.3%, demonstrating consistent transcriptional and translational regulation. FABP4 expression followed the same pattern, with mRNA and protein levels reduced by 23.5–31.4% and 17.9–26.7%, respectively, in a concentration-dependent manner. These results indicate that PGEs suppress adipogenesis- and lipid metabolism-related factors in a dose-dependent manner, thereby inhibiting adipocyte formation and contributing to their anti-obesity activity.

Similarly, as shown in Fig. 4E–H and Fig. 5E–H, PGE treatment significantly altered the expression of key regulators involved in energy metabolism, fatty acid synthesis, and lipid accumulation. SREBP-1c exhibited reduced transcriptional activity and translational efficiency, with mRNA and protein expression levels decreased by 6.3–24.9% and 4.3–22.8%, respectively, suggesting that PGE inhibits fatty acid biosynthetic pathways, thereby limiting lipid synthesis and accumulation. The lipogenic enzymes FAS and ACC exhibited consistent inhibitory trends, with mRNA expression reduced by 25.8–41.0% and 17.6–35.0% and protein expression decreased by 8.8–24.5% and 14.7–23.8%, respectively. These findings suggest that PGE disrupts the conversion of acetyl-CoA to malonyl-CoA and the subsequent saturated fatty acid synthesis, exerting a multitarget inhibitory effect on triglyceride accumulation [29]. In contrast, the expression of AMPK-α, a central regulator of cellular energy homeostasis, was elevated by 33.7–53.8% at the mRNA level and 29.2–47.9% at the protein level in the PGE-treated groups compared to the DC group. This upregulation indicates metabolic reprogramming via AMPK-α activation which promotes fatty acid oxidation while inhibiting lipid accumulation. Previous studies have shown that AMPK negatively regulates lipid metabolism by suppressing SREBP-1c activity, inducing ACC phosphorylation to decrease malonyl-CoA synthesis, and enhancing carnitine palmitoyltransferase 1 activity, thereby facilitating long-chain fatty acid entry into the β-oxidation pathway [30]. The observed effects in this study align with these roles of AMPK, supporting the hypothesis that PGEs exert anti-obesity effects by inhibiting lipid synthesis and enhancing fatty acid oxidation and metabolic activity at the molecular level.

In summary, PGEs demonstrated a consistent ability to inhibit both transcriptional and translational processes associated with adipocyte differentiation, fatty acid synthesis, and lipid accumulation in 3T3-L1 cells while concomitantly activating the AMPK-α signaling pathway to restore metabolic equilibrium. Specifically, the downregulation of key adipogenic transcription factors, including PPAR-γ, C/EBP-α, C/EBP-β, and FABP, together with the inhibition of fatty acid biosynthesis through the suppression of SREBP-1c, FAS, and ACC, offers strong evidence that PGE broadly inhibits intracellular lipid metabolism through multiple pathways [31]. Moreover, the significant upregulation of AMPK-α indicates its role as a central pathway mediator of PGE-induced fatty acid oxidation and overall anti-obesity activity. These findings reinforce the potential of plant-derived exosomes to influence obesity-related gene expression and bioactivity and provide foundational evidence for their development as functional food or pharmaceutical materials for the prevention and management of obesity. Future studies should further elucidate the underlying mechanisms by examining signaling pathways involving mTOR, leptin, and adiponectin, as well as insulin signaling, and validate the physiological effects of PGEs in in vivo models.

### Immunofluorescence analysis

To delineate the anti-obesity effects of PGEs, immunofluorescence staining of 3T3-L1 cells was performed using DAPI, MitoTracker, and F-actin to assess nuclear stability, mitochondrial activity, and cytoskeletal remodeling. Because adipogenic differentiation entails coordinated nuclear, metabolic, and structural reprogramming, these markers provide critical morphological readouts of PGE-mediated regulation [32]. Under adipogenic induction, PGE-treated groups were systematically compared with N.T. and DC groups to identify nuclear and organelle-level adaptations. Quantitative fluorescence data reflect reproducible patterns across independent experiments, with median fluorescence intensity (MFI) per cell applied to minimize outlier influence, thereby underscoring the robustness of PGE-mediated effects.

**Fig. 6:**
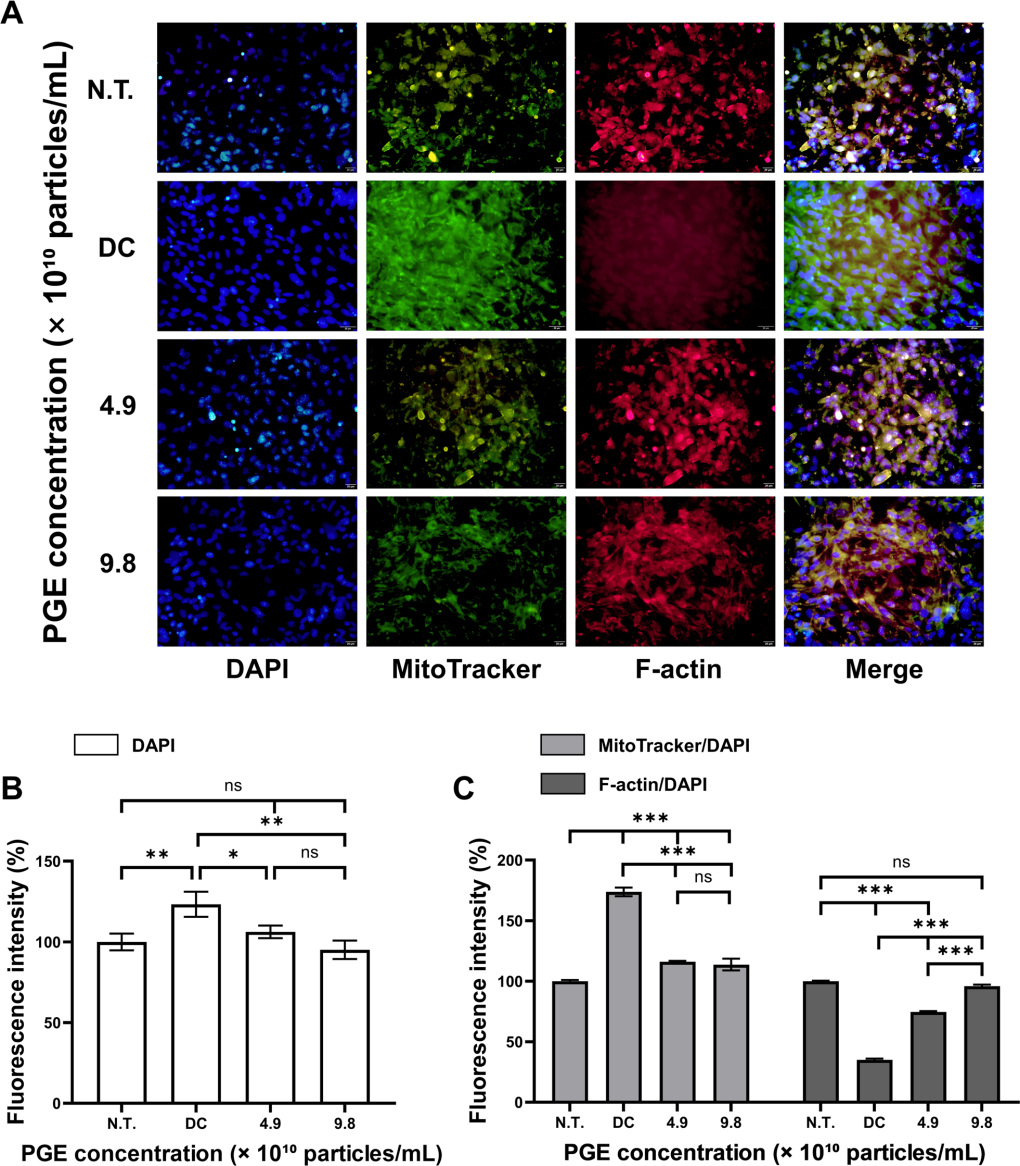
Immunofluorescence analysis of 3T3-L1 treated with *Panax ginseng-*derived exosomes. (**A**) Confocal images showing nuclei (DAPI, blue), MitoTracker (green), and F-actin (red). (**B**) Quantification of fluorescence intensity for DAPI. (**C**) Relative fluorescence intensity of MitoTracker and F-actin normalized to DAPI. Scale bars = 20 μm

As shown in Fig. 6, DAPI fluorescence increased to 123.3% in DC relative to N.T. (100.0%), consistent with nuclear architectural remodeling during adipogenic commitment. PGE treatment attenuated this increase, reducing nuclear intensity to 106.2% (4.9 × 10^10^ particles/mL) and 95.2% (9.8 × 10^10^ particles/mL), corresponding to 17.1% (*p* < 0.05) and 28.2% (*p* < 0.01) decreases versus DC. While DAPI normalization is a standard approach to control for cell number/nuclear content, we note that chromatin condensation itself can influence DAPI signal [33]. To mitigate this confound, intensities were extracted from nuclear masks generated by fixed thresholding across batches under identical exposure settings, and interpretation focuses on relative shifts; future work incorporating nuclear area, EdU/cell-cycle profiling, and chromatin accessibility (e.g., ATAC) will further distinguish DNA content from compaction effects [34]. Mitochondrial activity, quantified by MitoTracker/DAPI ratio, was markedly elevated in DC (173.8% of N.T.), aligning with metabolic activation during terminal adipogenesis. PGE significantly suppressed this activation to 116.1% and 113.8% in the 4.9 and 9.8 (10^10^ particles/mL) groups, reflecting 57.7% and 60.0% decreases versus DC (*p* < 0.001). Because signals were normalized to DAPI and extracted after background subtraction within cell-bounded masks, these reductions are unlikely to reflect mere proliferative expansion and instead indicate attenuation of genuine mitochondrial activation. Complementary functional assays (oxygen consumption rate, ATP production, mitochondrial membrane potential) will strengthen causal interpretation. In parallel, cytoskeletal remodeling assessed by F-actin/DAPI ratio revealed a pronounced reduction in DC (35.1% of N.T.), consistent with actin filament disassembly and loss of cortical tension during adipocyte rounding. PGE treatment restored cytoskeletal organization to 74.6% and 95.9% in the 4.9 and 9.8 (× 10^10^ particles/mL) PGE treated groups, corresponding to 39.5% and 61.0% gains versus DC (*p* < 0.001). Morphologically, PGE-treated cells exhibited enhanced cortical actin rings and aligned peripheral filaments, features associated with elevated cytoskeletal tension.

Mechanistically, the observed structural and metabolic corrections are consistent with AMPK-α activation, which suppresses lipogenesis by inhibiting ACC and downregulating adipogenic transcription factors while reinforcing cortical actin tension [35]. These molecular signatures—PPAR-γ, C/EBP-α/β, FABP4, SREBP-1c, FAS, and ACC downregulation with AMPK-α upregulation—collectively explain the reduced mitochondrial activation and restored F-actin architecture. Collectively, these fluorescence-based findings indicate that PGEs suppresses adipocyte differentiation by maintaining nuclear stability, attenuating mitochondrial activation, and reinforcing actin cytoskeletal integrity in an AMPK-centered manner [36]. To further bolster reproducibility, we employed constant acquisition parameters, per-cell segmentation, local background subtraction, batch controls, and reported both percentage change and MFI values. Future work integrating live-cell metabolic flux, traction force microscopy, and phospho-AMPK/ACC immunocytochemistry will be valuable for establishing causality and advance PGEs as an AMPK-mediated anti-adipogenic candidate.

### Analysis of AMPK-α expression

In this study, we quantitatively validated changes in AMPK-α expression at single-cell resolution during the differentiation of 3T3-L1 adipocytes, while simultaneously analyzing intracellular structural complexity (granularity) to objectively assess the degree of adipogenic differentiation. Adipocyte differentiation is not merely an increase in cell size but entails multidimensional cellular remodeling, including nuclear reorganization, metabolic activation of organelles, and cytoskeletal remodeling [37]. Therefore, a flow cytometry-based single-cell analysis that simultaneously evaluates AMPK-α expression patterns and structural features provides a critical approach to elucidating the anti-obesity mechanism of PGEs [38].

**Fig. 7:**
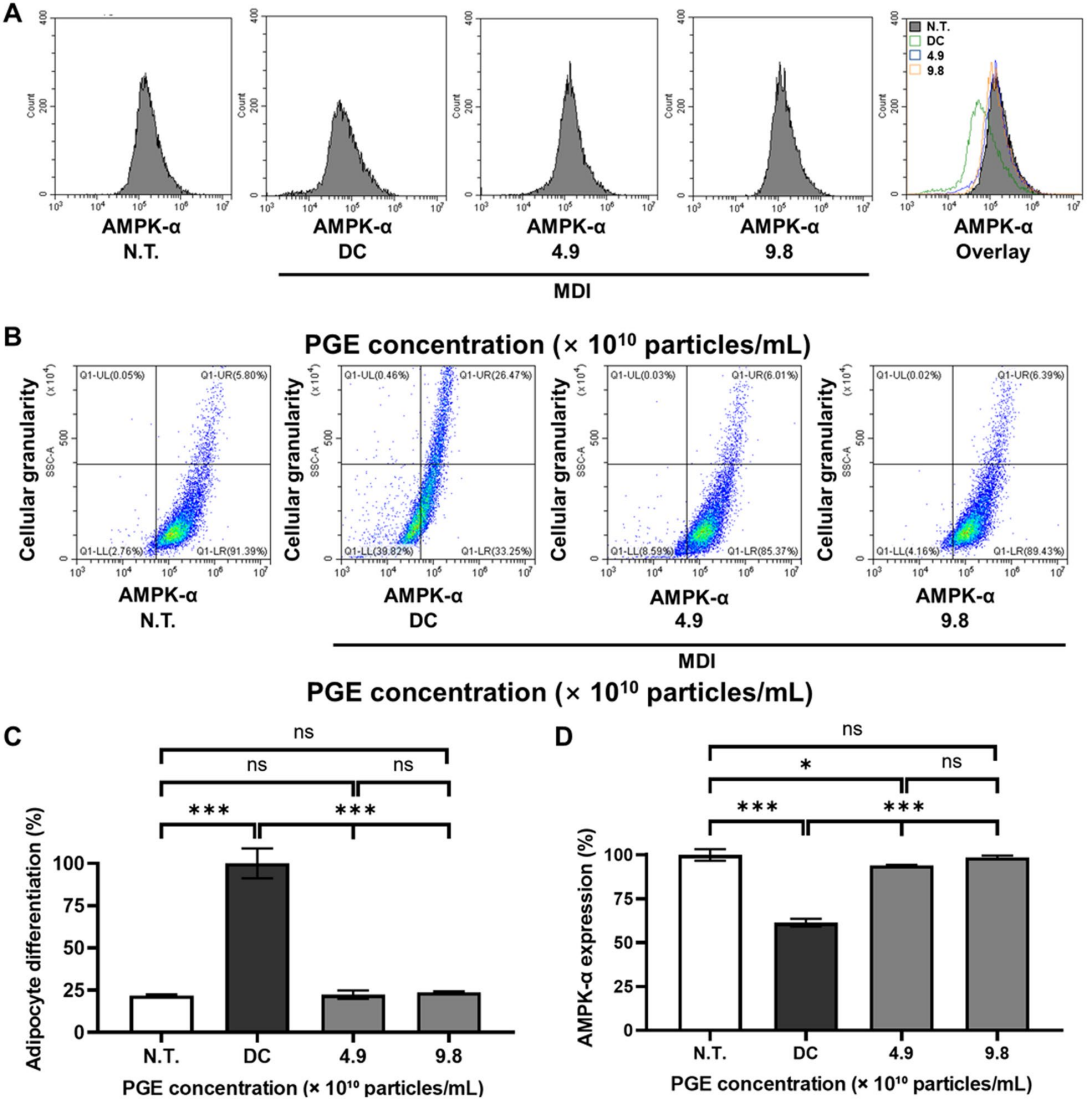
Effects of *Panax ginseng-*derived exosomes (PGE) on AMPK-α expression and adipocyte differentiation in 3T3-L1 cells. (**A**) Flow cytometry histograms showing AMPK-α fluorescence intensity in N.T., DC, and PGE-treated groups. (**B**) Representative dot plots indicating changes in AMPK-α expression versus cellular granularity. (**C**) Quantification of adipocyte differentiation normalized to DC (100%). (**D**) Quantification of AMPK-α expression normalized to N.T

Flow cytometric analysis demonstrated that when normalized to 100 for the DC group, the N.T. group showed only 21.8%, confirming markedly reduced adipogenic progression (Fig. 7). By contrast, PGE treatment resulted in 22.3% and 23.7% at 4.9 × 10^1^^0^ and 9.8 × 10^1^^0^ particles/mL, respectively, corresponding to 76.2–77.7% decreases compared with DC. These findings indicate that PGE effectively suppresses the increase in structural complexity associated with adipogenic induction and maintains stable inhibitory effects on differentiation even at higher concentrations. Thus, PGE can be interpreted as modulating the intracellular remodeling rate throughout the adipogenic process, thereby contributing to the inhibition of adipocyte differentiation.

**Table 2:** Exosome recovery and concentration at each processing step during the purification of Panax ginseng-derived exosomes

Processing step	Volume (mL)	Relative volume (%)	Concentration factor	Concentration (particles/mL)	Normalized exosomes yield (%)
Preparation of ginseng slurry	8,821.3	100.0	1.0	–	–
Primary low-speed centrifugation	6,340.4	71.9	1.4	–	–
Secondary high-speed centrifugation	6,094.1	69.8	1.5	–	–
Primary microfiltration (1.22 μm membrane)	6,013.4	68.2	1.5	–	–
Secondary microfiltration (0.44 μm membrane)	5,942.7	67.4	1.5	–	–
Tertiary microfiltration (0.22 μm membrane)	5,876.4	66.6	1.5	5.1 × 10^11^	100.0
TFF Concentrate (retentate)	58.6	0.7	150.5	3.9 × 10^12^	771.2
TFF permeate (filtrate)	5607.2	63.6	–	9.3 × 10^9^	1.7
TFF wash buffer (diafiltration Buffer)	94.7	1.1	–	3.3 × 10^11^	1.0

Analysis of AMPK-α expression revealed that, with the N.T. group set to 100%, the DC group decreased to 61.4%, reflecting a 38.6% reduction in AMPK activity during adipogenic progression. However, in the PGE-treated groups, AMPK-α expression was significantly restored in a dose-dependent manner, reaching 94.0% at 4.9 × 10^1^^0^ particles/mL and 98.6% at 9.8 × 10^1^^0^ particles/mL, corresponding to 32.6% and 37.2% increases relative to DC. These results suggest that PGE effectively reverses the adipogenesis-associated suppression of cellular energy-sensing pathways and may be mechanistically linked to the inhibition of fatty acid synthesis via ACC suppression, as well as reduced lipid accumulation through downregulation of FAS and SREBP-1c [39].

Collectively, this study quantitatively demonstrates that PGE suppresses adipocyte differentiation while restoring AMPK-α expression at the single-cell level, thereby contributing to the normalization of metabolic balance. This finding suggests that PGE is not merely an auxiliary factor but has possesses the potential to attenuate lipid accumulation by regulating the expression of lipogenic genes through AMPK pathway engagement. Furthermore, these results are consistent with the activation patterns of the AMPK pathway observed in preceding previous Western blot analyses, thereby strengthening the reliability of the findings through cross-validation. Accordingly, this study provides molecular and cellular evidence supporting the potential development of PGE as a functional anti-obesity agent mediated by AMPK signaling regulation.

### Quantitative and qualitative analysis of ginsenosides

To elucidate the molecular mechanisms underlying the anti-obesity effects of PGEs, it is crucial to confirm and quantify the presence levels of bioactive constituents within the exosomal cargo. Ginsenosides, a class of saponin-type phytochemicals abundantly present in *P. ginseng*, are well-documented for their anti-obesity properties through multiple mechanisms, including activation of the AMPK signaling pathway, inhibition of adipocyte differentiation, modulation of lipid metabolism, and anti-inflammatory and antioxidant activities [40]. Accordingly, in this study, the ginsenoside composition and concentration in PGEs were analyzed using a targeted liquid chromatography–tandem mass spectrometry (LC–MS/MS) approach.

The analysis identified three predominant ginsenosides—Rg1, Re, and Rb1—within PGEs. Their retention times were recorded at 2.34 min for Rg1, 2.31 min for Re, and 9.84 min for Rb1, showing strong concordance with those of authentic standards (Fig. S1). Quantitative profiling (Table 3) indicated that Rg1 was the most abundant constituent (0.19 mg/kg), followed by Re (0.16 mg/kg) and Rb1 (0.07 mg/kg). These findings indicate that PGEs function as a composite bioactive formulation rather than a single-compound delivery system, with ginsenosides likely acting as key molecular modulators of adipogenesis and lipid biosynthesis. Previous studies have demonstrated that Rg1 suppresses adipogenic transcription factors, including PPAR-γ and members of the C/EBP family, through AMPK activation while simultaneously downregulating lipogenic enzymes to attenuate lipid accumulation [41]. In the present study, the observed elevation of AMPK-α expression and decreased lipid deposition in PGE-treated cells are consistent with with these mechanistic insights, suggesting the potential of Rg1 as a principal contributor to the observed molecular responses.

**Table 3:** Retention time and multiple reaction monitoring results for identification of ginsenoside in *Panax ginseng*-derived exosomes

Compound Name	Retention time (min)	Formula	Moleculur weight	Fragment (m/z)	Concentration (mg/kg)
Ginsenoside Rg1	2.34	C_48_H_82_O_18_	801.02	639.45, 477.48	0.19
Ginsenoside Re	2.31	C_42_H_72_O_14_	947.17	367.18, 791.42promoting	0.16
Ginsenoside Rb1	9.84	C_42_H_72_O_14_	1109.30	182.07, 947.52	0.07

Furthermore, the ginsenosides within PGEs are likely to act synergistically to activate AMPK-α signaling, thereby restoring energy homeostasis and suppressing both adipocyte differentiation and lipid biosynthesis. AMPK activation downregulates fatty acid synthesis by phosphorylating ACC, reducing malonyl-CoA levels, and diminishing the transcriptional activity of SREBP-1c, thereby limiting triglyceride storage [42]. These molecular mechanisms are consistent with the phenotypic changes observed in PGE-treated  cells, including decreased lipid accumulation, reduced cell size, and downregulated FABP4 expression. Notably, the present study was designed to characterize the composite bioactivity of PGEs, without performing direct comparative assays using isolated ginsenosides. Future research should evaluate the anti-obesity efficacy and signaling pathway modulation of PGEs versus individual ginsenosides (Rg1, Re, and Rb1), both independently and in combination, to quantitatively determine the contribution of each component. Such investigations will not only refine mechanistic interpretation but also provide a scientific basis for optimizing nutraceutical or pharmaceutical strategies based on plant-derived exosomes for obesity prevention and metabolic regulation.

## Conclusion

Obesity is not merely an increase in body weight; it represents a complex metabolic dysregulation characterized by enhanced adipocyte differentiation and fatty acid accumulation. Growing attention has been directed toward plant-derived functional materials capable of modulating adipogenic and lipogenic pathways, with exosomes emerging as promising next-generation delivery systems that transport diverse bioactive molecules in a stable manner. In this study, we evaluated the metabolic regulatory potential of PGEs by assessing their effects on transcriptional and translational regulators of adipocyte differentiation and fatty acid synthesis in 3T3-L1 cells. Specifically, we analyzed the expression of adipogenesis-related transcription factors (PPAR-γ, C/EBP-α, C/EBP-β, and FABP4) and lipogenesis-associated regulators (SREBP-1c, FAS, and ACC) in relation to AMPK-α activation, and evaluated their impact on cellular morphology, including mitochondrial function and cytoskeletal organization.

PGE treatment significantly downregulated PPAR-γ and C/EBP-α, the principal drivers of adipogenesis, indicating potential inhibition of preadipocyte-to-adipocyte differentiation. The suppression of C/EBP-β, an early-stage regulator, and FABP4, a late-stage adipogenic marker, further indicates regulation across multiple stages of adipogenesis. Concurrently, the expression of SREBP-1c, FAS, and ACC—central regulators of de novo fatty acid biosynthesis and storage—was markedly reduced, aligning with the inhibition of lipid anabolism. Notably, AMPK-α upregulation indicates the activation of a pivotal metabolic regulator that indirectly suppresses ACC via phosphorylation, thereby promoting fatty acid oxidation. Immunofluorescence analysis revealed alterations in mitochondrial activity and F-actin cytoskeletal organization in PGE-treated cells, suggesting that the anti-adipogenic effects extend beyond transcriptional regulation to structural remodeling of energy systems and cellular architecture. These morphological findings corroborate the observed molecular changes. Furthermore, LC–MS/MS profiling confirmed the presence of multiple ginsenosides (Rg1, Re, and Rb1) within PGEs, which likely act synergistically in metabolic regulation, warranting further mechanistic investigation.

Collectively, our findings demonstrate that PGEs simultaneously modulate adipogenic and lipogenic pathways through multitarget regulation within the AMPK-α–centered metabolic network, thereby contributing to lipid metabolic homeostasis. This study underscores the biological potential of plant-derived exosomes as functional delivery platforms, representing a novel strategy that complements and surpasses conventional single-compound approaches. The present study was limited to in vitro analyses using the 3T3-L1 preadipocyte model, a well-established system for investigating adipogenesis and lipid metabolism. To overcome the current limitation in exosome yield, we plan to establish a scalable exosome production system, which will enable subsequent in vivo animal studies to validate the physiological relevance and translational potential of PGEs in future research. Nonetheless, this study is limited to quantitative molecular analyses; thus, future research should include the assessment of AMPK and ACC phosphorylation status, isolation and functional validation of individual cargo components, and in vivo efficacy studies to confirm translational potential. Such investigations will clarify the mechanistic basis, safety, and application scope of PGEs, ultimately advancing the academic and industrial value of natural metabolic modulators based on plant-derived exosomes.

## Supplementary Information

Supplementary Material 1.

## Data Availability

The datasets generated during and/or analysed during the current study are available from the corresponding author on reasonable request.
