# Supplementary material for: Anti-obesity Effects of Panax ginseng-derived exosomes via AMPK-mediated inhibition of adipocyte differentiation and lipogenesis
Source: Nat Prod Bioprospect. 2026 Jan 9;16(1):8. doi: 10.1007/s13659-025-00561-4 (PMC12783488; doi:10.1007/s13659-025-00561-4)
Supplement: Supplementary file 1 — Supplementary Material 1. [file 13659_2025_561_MOESM1_ESM.docx]

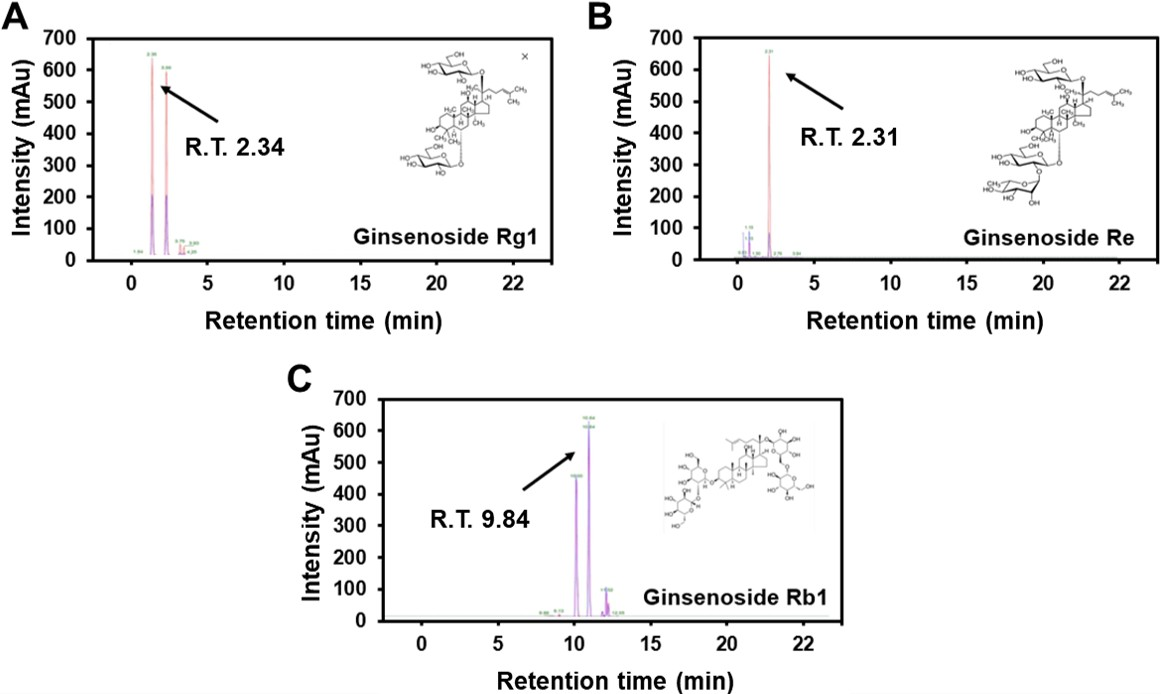
 **Fig. S1** Quantitative and qualitative analysis of main compounds in *Panax ginseng*-derived exosome based on retention time and mass-to-charge (m/z) using LC-MS/MS. Relative abundance is represented as ion signal intensity in relation to total ion intensity within spectrum. (A) ginsenoside Rg1, (B) Re and (C) Rb1
